# Draft Genome Sequence of Yokenella regensburgei Strain WCD67, Isolated from the Boxelder Bug

**DOI:** 10.1128/MRA.00104-20

**Published:** 2020-04-09

**Authors:** Desiree J. Meyers, Brittany A. Leigh, Marcel Huntemann, Alicia Clum, Stephan Ritter, Krishnaveni Palaniappan, I-Min Chen, Dimitrios Stamatis, T. B. K. Reddy, Ronan O’Malley, Chris Daum, Nicole Shapiro, Natalia Ivanova, Nikos C. Kyrpides, Tanja Woyke, William B. Whitman

**Affiliations:** a Department of Biology, Wayne County Community College District, Detroit, Michigan, USA; b Department of Biological Sciences, Vanderbilt University, Nashville, Tennessee, USA; c Joint Genome Institute, U.S. Department of Energy, Berkeley, California, USA; d University of Georgia, Department of Microbiology, Athens, Georgia, USA; Indiana University, Bloomington

## Abstract

We report here the draft genome sequence of Yokenella regensburgei strain WCD67, isolated from the boxelder bug (Boisea trivittata). The genome is 5,277,883 bp in size, has a GC content of 54.12%, and has 5,416 genes. A total of 17 mobile elements were discovered, 6 of which were predicted to be phages.

## ANNOUNCEMENT

Yokenella regensburgei, a Gram-negative member of the *Enterobacteriaceae* family, has previously been isolated from European firebugs, stink bugs, stick insects, and termites (1–5). Here, we report the isolation of *Y. regensburgei* strain WCD67 from an additional host insect, the boxelder bug (Boisea trivittata), along with its whole-genome sequence. Insects often maintain beneficial partnerships with microorganisms which possess factors that ultimately influence insect development and fitness (6), and the genome sequence of *Y. regensburgei* WCD67 may help to elucidate the genomic basis of symbiosis with its insect host. Further, the genome sequence of WCD67 will extend our understanding of the *Y. regensburgei* pangenome, especially since insect symbionts such as *Yokenella* spp. have biotechnological applications through conversion of biomass to biofuel (7).

WCD67 was isolated from a boxelder bug in southeastern Michigan (global positioning system [GPS] coordinates, 42.214610, −83.242693) in April 2018. Within 2 h of collection, the boxelder bug was euthanized by freezing at −20°C for 10 min and surface sterilized (8) before it was aseptically dissected to retrieve the gut. The extracted gut was washed in sterile nutrient broth and homogenized, and the homogenate was used to inoculate nutrient agar (NA) medium that was incubated at 37°C for 48 h. A nonpigmented, Gram-negative, motile bacillus growing on the NA was subcultured, and a pure colony was used for 16S rRNA gene sequencing (Genewiz, South Plainfield, NJ). BLAST analysis of the sequenced 16S rRNA amplicon (GenBank accession number MN047528) yielded a 99.39% match with the type strain of *Y. regensburgei*, ATCC 49455^T^ (i.e., CIP 105435 or NBRC 102600).

Total genomic DNA was isolated from a single colony grown overnight at 37°C on Trypticase soy agar using a modified phenol-chloroform method (https://1ofdmq2n8tc36m6i46scovo2e-wpengine.netdna-ssl.com/wp-content/uploads/2014/02/JGI-Bacterial-DNA-isolation-CTAB-Protocol-2012.pdf). DNA was quantified using a Qubit fluorometer and further quality checked with gel electrophoresis before whole-genome sequencing was performed by the U.S. Department of Energy Joint Genome Institute (JGI) in Berkeley, California. Using a Kapa Biosystems library preparation kit, 300-bp fragments were end repaired, A tailed, and ligated with adapters. The prepared library was amplified using a Kappa Biosystems library quantitative PCR (qPCR) kit and subsequently sequenced using the Illumina NovaSeq 6000 platform, which generated 17,704,142 paired-end (2 × 151-bp) reads totaling 2,673,325,442 bp. Raw Illumina sequence data were quality filtered using BBTools (9). The following steps were then performed for assembly: (i) artifact-filtered and normalized Illumina reads were assembled using SPAdes v3.13.0 (–phred offset 33, –cov cutoff auto, –t 16, –m 64, –careful, –k 25,55,95) (10); and (ii) contigs were discarded if the length was <1 kb (BBTools reformat.sh: minlength). The final draft assembly was based on 1,498,759,959 bp of Illumina data with a mapped coverage of 297.9× and contained 103 contigs (*N*/*L*_50_, 8/222,255 bp) in 97 scaffolds (*N*/*L*_50_, 8/222,255 bp). The genome has 5,277,883 bp and a GC content of 54.12%. Whole-genome comparisons were determined using the compare genome analysis function in the JGI Integrated Microbial Genomes and Microbiomes (IMG/M) pipeline (11). The average nucleotide identity (ANI) between *Y. regensburgei* strains WCD67 and ATCC 49455^T^, determined using a pairwise alignment method, is 99.152%.

Genome annotation of WCD67 was performed in the IMG/M pipeline (11). The genome contains 5,214 protein-coding genes, 157 RNA genes, and 45 genes classified as regulatory or miscellaneous genes, to make up a total of 5,416 genes. Some of the enzymes encoded are cellulase, beta-glucosidase, esterase, and glutathione *S*-transferase. The RNA genes comprised 5 rRNA genes, 78 tRNA genes, and 74 noncoding RNA genes. A total of 17 mobile genetic elements were discovered within the *Y. regensburgei* WCD67 draft genome sequence using VirSorter v1.0.3 (12). Six of these mobile elements were predicted to be phages (category 2), including one complete circular prophage (28,360 bp) similar to other *Enterobacteriaceae* phages.

### Data availability.

This whole-genome shotgun project has been deposited at DDBJ/ENA/GenBank under the accession number JAABVJ000000000. The version described in this paper is version JAABVJ010000000. The project data have been submitted under BioProject accession number PRJNA546626, and the raw sequences have been submitted under SRA accession number SRX6876986.
